# Learning from Ebola Alerts in the Pandemic Agreement Era: Preparedness, Trust, and Shared Responsibility

**DOI:** 10.3390/healthcare14172712

**Published:** 2026-08-25

**Authors:** Marco Dettori

**Affiliations:** Department of Medicine, Surgery and Pharmacy, University of Sassari, 07100 Sassari, Italy; madettori@uniss.it

**Keywords:** Ebola disease, Bundibugyo virus, Pandemic Agreement, preparedness, risk communication, community trust, global health security, infection prevention and control, health systems, public health emergencies

## Abstract

Ebola alerts reveal the truth about preparedness. They show whether a health system can recognize danger early, protect health workers, isolate and care for patients, trace contacts, communicate clearly, and sustain public trust before fear replaces cooperation. In the Pandemic Agreement Era, this truth has become harder to ignore. The 2026 Bundibugyo virus disease outbreak in the Democratic Republic of the Congo and Uganda is a sharp reminder that the principles of pandemic prevention, preparedness, response, equity, and cooperation become meaningful only when they work where the first cases occur. In the absence of licensed vaccines and specific treatments for Bundibugyo virus disease, preparedness depends on the basic functions of public health: surveillance, diagnosis, infection prevention and control, referral, risk communication, community engagement, and operational coordination. These functions are often described as routine. They are not. During high-consequence infectious disease alerts, they serve as the primary protective infrastructure. Ebola also exposes a deeper ethical failure. Frontline countries and communities are expected to contain threats of global relevance, but international support often intensifies only after the risk becomes visible beyond the affected area. This reactive model is neither fair nor efficient. The WHO Pandemic Agreement defines a political direction for a more equitable and coordinated global response. Here, Ebola is not presented as a typical pandemic event but as an operational test of preparedness, solidarity, early support to affected settings, and international cooperation. Ebola alerts test whether that direction can become an operational reality before escalation. Digital tools, telemedicine, and artificial intelligence may strengthen this agenda but only when they make local systems faster, safer, more trusted, and more usable. Ebola preparedness is therefore not only a technical task. It is a test of responsibility.

## 1. Introduction

### 1.1. Ebola Tells the Truth About Preparedness

Ebola is a severe infectious disease. It is also a mirror.

It shows whether public health systems can act before uncertainty becomes panic. It shows whether health workers are protected or left exposed. It shows whether communities trust institutions enough to report symptoms, accept isolation, cooperate with contact tracing, and change burial practices. It shows whether international solidarity begins early or waits until the outbreak threatens wider interests.

This is why Ebola alerts matter even when case numbers remain geographically limited. They are not only local crises. They are tests of preparedness.

The 2026 outbreak of Ebola disease caused by the Bundibugyo virus in the Democratic Republic of the Congo and Uganda brought these issues back into view. Public reports described a rapidly evolving outbreak, cross-border response needs, delayed detection, diagnostic challenges, shortages of protective equipment, infected health workers, mistrust affecting response activities, and the absence of approved vaccines or treatments for Bundibugyo virus disease [1,2,3,4,5]. The event emerged just after the World Health Assembly adopted the WHO Pandemic Agreement, which was designed to strengthen pandemic prevention, preparedness and response, and to make future responses more equitable and coordinated [6]. This timing matters. Although Ebola outbreaks are usually localized or regional rather than typical pandemic events, they give the new global preparedness agenda an operational face by testing whether its principles can work where the first cases occur.

The event combined several hard conditions: severe disease, affected health workers, delayed detection in some settings, difficult contact tracing, community mistrust, insecurity, and limited strain-specific biomedical countermeasures [1,2,3]. For Zaire ebolavirus disease, vaccines and therapeutics have changed the response landscape [7,8]. For Bundibugyo virus disease, licensed vaccines and specific treatments are not currently available [1,3]. Under these conditions, the old public health functions become decisive again.

This is the uncomfortable lesson. When biomedical rescue is limited, preparedness is not a slogan. It is the system itself.

This Viewpoint addresses a specific gap: while Ebola preparedness has been widely discussed in relation to outbreak control, response capacity, and biomedical countermeasures, the implications of the newly adopted WHO Pandemic Agreement for localized, high-consequence outbreaks remain less explicit. Its purpose is to interpret the 2026 Bundibugyo virus disease outbreak as an operational test of preparedness, equity, trust, and early international support.

### 1.2. The Strongest Systems Make Early Action Ordinary

Preparedness is often described through plans, protocols, simulations, and emergency committees. Ebola shows whether these instruments work in real life.

A suspected case must be recognized quickly. A health worker must know what to do. A patient must be isolated without being abandoned. A sample must reach a laboratory. Results must return fast enough to guide action. Contacts must be identified, followed, and supported. Communities must understand why measures are being taken. Health facilities must continue to function without becoming transmission sites.

If one of these steps fails, the others weaken.

This is why Ebola preparedness cannot be reduced to outbreak response after confirmation. The decisive period often starts earlier: when symptoms are still ambiguous, when fear is still manageable, when trust can still be preserved, and when a small delay can become a transmission chain.

The strongest systems are not those that react dramatically. They are those that make early action ordinary.

### 1.3. Basic Public Health Functions Are the Real Protective Infrastructure

In high-income debates, preparedness is often associated with innovation: vaccines, therapeutics, predictive models, digital surveillance, artificial intelligence, and genomic intelligence. These tools matter. But Ebola reminds us that the first infrastructure of preparedness is more basic.

It is a trained nurse recognizing a risk.

It is a protected clinician who does not become the next case.

It is a functioning referral pathway.

It is a laboratory network that does not lose days.

It is a community leader who can explain why isolation protects families.

It is a contact tracer who is trusted enough to enter a household.

It is a burial team that protects life without humiliating grief.

These are not secondary details. They are the core of outbreak control.

When specific vaccines and treatments are unavailable, these functions carry even more weight [1,3]. Supportive care, infection prevention and control, safe triage, laboratory confirmation, contact tracing, risk communication, and community engagement become the real protective infrastructure [9,10,11].

This infrastructure is fragile if it is built only during emergencies. It requires trained personnel, supplies, trust, transport, local knowledge, communication channels, and institutional memory before the alert arrives.

### 1.4. Trust Is Not a Soft Variable

Ebola response fails when people hide symptoms, avoid facilities, reject burial teams, distrust contact tracing, or believe that isolation means disappearance. These behaviours are sometimes described as “resistance”. That word is too easy. Often, they are rational responses to fear, past harm, poor communication, social stigma, economic pressure, and weak institutional credibility. Evidence from the 2022–2023 Ebola response in Uganda similarly identified delayed consultation, poor communication, misinformation, limited support for community workers, and coordination failures as barriers to effective community engagement [12].

Trust is not decorative. It is operational.

Without trust, surveillance becomes blind. Contact tracing becomes negotiation. Isolation becomes coercion. Risk communication becomes noise. Health workers become targets of suspicion. Rumours move faster than response teams.

Good risk communication does not mean reassuring people vaguely. It means telling the truth early. It means explaining what is known, what is uncertain, what people should do, and what the system will do for them. It means avoiding both panic and minimization. It means speaking through voices that communities already recognize. Consistently, the 2026 IHR temporary recommendations identify risk communication and community engagement, community-based surveillance, local coordination, and safe and dignified burial practices as operational components of the Bundibugyo virus disease response [13].

This is not new. Historical experience from the 1918 influenza pandemic in Italy shows that even technically reasonable infection-control measures can fail when they collide with social habits, rituals, mistrust, and weak public acceptance [14].

Digital environments make this harder. Public reactions to health threats can be amplified by media cycles, social platforms, anger, fear, and misinformation [15]. This is not separate from outbreak control. It is part of the field. In the digital era, Ebola preparedness must include listening systems, rumour management, trusted local communication, and rapid correction of harmful narratives.

The question is not whether people receive information. The question is whether they believe it in time to act.

### 1.5. Global Protection Begins Where the First Cases Occur

The Pandemic Agreement was adopted because COVID-19 exposed gaps and inequities in national and global responses. WHO describes the agreement as a international legal instrument adopted under Article 19 of the WHO Constitution intended to strengthen international collaboration in pandemic prevention, preparedness and response, built on principles of equity, solidarity, and respect for national sovereignty. It also highlights surveillance, a One Health approach, stronger health systems, the health and care workforce, public communication, international cooperation, sustainable financing, and more equitable access to pandemic-related health products [6]. Ebola translates those principles into the field. This does not mean treating Ebola as a conventional pandemic scenario. Rather, Ebola alerts show whether the principles of preparedness, solidarity, equity, and early international support can be applied in localized or regional emergencies before escalation. It shows what equity, solidarity, and preparedness mean before a threat becomes internationally disruptive.

The Agreement’s relevance becomes more concrete through mechanisms intended to connect early detection with equitable support. The Pathogen Access and Benefit-Sharing system is designed to pair the rapid sharing of pathogens and sequence information with fair and timely access to resulting vaccines, diagnostics, and therapeutics, while the Coordinating Financial Mechanism is intended to strengthen preparedness capacities and support surge financing, particularly in developing countries [16,17]. For Ebola-prone settings, these mechanisms will matter only if they translate into earlier laboratory support, protected health workers, resilient supply chains, and financing before escalation. Previous analyses of post-COVID health-system resilience and Ebola preparedness have emphasized capacities that conventional preparedness assessments may overlook [18,19]. This Viewpoint extends that literature by examining how a current localized Ebola outbreak tests the equity, trust, and early-support commitments of the newly adopted Pandemic Agreement.

The International Health Regulations (2005) provide the existing legally binding framework for surveillance, notification to WHO, information sharing, cross-border coordination, and the maintenance of national core capacities for detection and response [20]. The WHO Pandemic Agreement should therefore be understood as complementary to, rather than a substitute for, the IHR. In the present outbreak, immediate operational requirements are anchored principally in the IHR framework and in the temporary recommendations issued under it [13,20], whereas the Pandemic Agreement adds a broader policy architecture centred on equity, sustainable financing, international cooperation, and future pathogen access and benefit-sharing. Its current operational implications should not be overstated: although the Agreement was adopted in 2025, as of August 2026 the PABS annex remains under negotiation and the full Agreement is not yet open for signature, ratification, or implementation [16,17].

Ebola outbreaks usually begin where health systems already face pressure: limited infrastructure, workforce shortages, difficult geography, insecurity, poverty, displacement, or weak service access. These are the places expected to detect, contain, communicate, and absorb threats that the world later describes as matters of global health security.

This is the central injustice.

Frontline communities are often treated as the first wall of global protection, but too often receive full international attention only after the wall begins to crack. The Pandemic Agreement points in the opposite direction. It recognizes that global protection cannot be separated from earlier, fairer and more coordinated support where risks first emerge [6].

This is not only an ethical point. It is operational. Waiting for escalation wastes time. It also shifts the cost of global safety onto local systems that may already be under-resourced. If Ebola containment protects the wider world, then local preparedness is not a local luxury. It is the first layer of global health security.

Support should arrive before headlines. Laboratories, personal protective equipment, workforce training, safe transport, surveillance, community engagement, primary care continuity, and social support should not depend on an international alarm. They should be part of a sustained preparedness investment.

Risks do not spread in empty space. They move through settlements, transport systems, health services, workplaces, families, and communication networks. Place matters. Infrastructure matters. Trust matters. The Pandemic Agreement defines the direction. Ebola alerts test whether that direction can become an operational reality.

### 1.6. Technology Is Useful Only if It Improves Action

Digital tools, telemedicine, and artificial intelligence can strengthen Ebola preparedness. They can support event-based surveillance, laboratory reporting, contact follow-up, logistics, remote training, clinical consultation, social listening, and message adaptation.

But technology is useful only if it improves action.

A dashboard is not preparedness if data are incomplete. A model is not response if no team can act on its signal. A chatbot is not risk communication if people do not trust the institution behind it. Social listening is not community engagement if nobody responds to what is heard. Telemedicine is not capacity building if it leaves local workers unsupported after the emergency.

The right question is simple: does the tool make the response faster, safer, clearer, and more trusted?

If the answer is no, it is not preparedness. It is decoration.

This matters because public health is entering an era in which AI and digital systems are often presented as solutions before the problem is properly defined. WHO guidance on artificial intelligence for health stresses that ethics, governance, human rights, and public trust should guide the design and use of these systems [21]. Ebola reverses the logic. The problem is concrete: detect early, protect workers, care safely, trace contacts, communicate truthfully, support communities, and coordinate under pressure. Technology has value only when it strengthens these functions.

These potential applications should not be read as evidence of proven effectiveness in every setting. Their implementation remains constrained by connectivity, data quality, interoperability, workforce capacity, governance, privacy, and trust; in this Viewpoint, they are therefore presented as operational possibilities that require context-specific evaluation rather than as established solutions [21].

### 1.7. Ebola Keeps Repeating the Same Lessons Because Systems Keep Forgetting Them

Ebola preparedness can be summarized in a few hard lessons.

First, alerts matter before they become crises. The time to protect health workers, test reporting channels, prepare messages, and strengthen referral pathways is before the first suspected case appears.

Second, basic public health functions are not basic in practice. They require people, equipment, transport, laboratories, training, supervision, and trust.

Third, communities are not obstacles to response. They are part of response. Measures that ignore fear, grief, stigma, and local authority will fail.

Fourth, global health security begins locally. Supporting affected settings early is both ethical and self-protective.

Fifth, digital tools and artificial intelligence should be judged by whether they strengthen local capacity not by whether they appear innovative.

These lessons are not new. That is precisely the problem. Ebola keeps repeating them because systems keep forgetting them between emergencies.

### 1.8. The Pandemic Agreement Sets the Direction, but Ebola Tests the Reality

The operational test is whether health systems are ready before a crisis becomes visible, communication preserves trust, health workers are protected, communities are treated as partners, and frontline settings receive support before fear travels.

The 2026 Bundibugyo virus disease outbreak is not only another Ebola event. It is a reminder that preparedness cannot depend on biomedical countermeasures alone. When specific vaccines and treatments are limited, the essential functions of public health become the main line of defence.

The Pandemic Agreement sets a political and institutional direction for a more equitable and coordinated approach to pandemic prevention, preparedness and response. Ebola alerts test whether that direction can become real before escalation.

The deeper lesson is political and ethical. Local systems should not be left alone to contain threats of global relevance. Ebola preparedness is a shared responsibility before escalation, not a gesture of concern after it.

The next Ebola alert will ask the same question again: did we prepare early enough, clearly enough, fairly enough, and with enough trust to act before fear took control?

Where possible, outbreak-related claims were cross-checked across WHO institutional reports and contemporaneous reporting, while peer-reviewed literature was used to support broader claims concerning preparedness, health-system resilience, community engagement, and risk communication. These interpretations should be read in light of the rapidly evolving outbreak and the policy-oriented nature of this Viewpoint. The discussion is an expert policy interpretation supported by selected peer-reviewed studies, WHO guidance, official outbreak reports, and contemporaneous reporting chosen for their direct relevance, recency, institutional authority, and applicability to the 2026 outbreak and the Pandemic Agreement. Official reports provide authoritative epidemiological and operational information but are updated as evidence changes, while contemporaneous journalism may capture field conditions that are not yet represented in peer-reviewed literature. The article therefore does not claim causal inference or generalizability from a single outbreak; it uses the available evidence to develop a policy interpretation whose relevance should be reassessed as the outbreak and implementation of the Pandemic Agreement evolve [4,5,18,19].

Accordingly, the policy implications developed from this single outbreak should not be generalized automatically to other Ebola outbreaks or to emerging infectious disease emergencies. Their transferability depends on pathogen characteristics, health-system capacity, sociopolitical and security conditions, cross-border dynamics, and the stage of the emergency response.

## 2. Conclusions

Ebola alerts illustrate that preparedness is measured not by plans or technologies alone but by the capacity to detect risk early, protect health workers, maintain essential public health functions, communicate transparently, and sustain community trust. Where strain-specific vaccines and treatments are unavailable, these capacities become the principal protective infrastructure and must be strengthened before an emergency begins.

From the policy perspective advanced in this Viewpoint, in the Pandemic Agreement era, early and equitable support to affected settings should be understood as both an ethical obligation and a practical condition of global health security. The central lesson is therefore clear: Ebola preparedness must be sustained locally, supported internationally, and judged by whether systems can translate solidarity, evidence, and innovation into timely action before escalation.

Operationally, national authorities should maintain surveillance, laboratory readiness, infection prevention and control, timely notification, and continuity of essential services; regional bodies should strengthen cross-border coordination and shared readiness; the WHO and other international partners should provide early technical, logistical, and financing support; and frontline public health actors should be equipped to sustain case detection, contact tracing, risk communication, and community engagement [13,20].

## Data Availability

No new data were generated or analyzed for this Viewpoint. All sources discussed in this article are cited in the reference list.
